# Identification of Copy Number Variants Defining Genomic Differences among Major Human Groups

**DOI:** 10.1371/journal.pone.0007230

**Published:** 2009-09-30

**Authors:** Lluís Armengol, Sergi Villatoro, Juan R. González, Lorena Pantano, Manel García-Aragonés, Raquel Rabionet, Mario Cáceres, Xavier Estivill

**Affiliations:** 1 Genetic Causes of Disease Group, Genes and Disease Program, Center for Genomic Regulation (CRG-UPF) and CIBERESP, Barcelona, Catalonia, Spain; 2 Quantitative Genomic Medicine Laboratories (qGenomics), Barcelona, Catalonia, Spain; 3 Center for Research in Environmental Epidemiology (CREAL), Barcelona, Catalonia, Spain; 4 Genetics Unit, Department of Health and Experimental Life Sciences, Pompeu Fabra University (UPF), Barcelona, Catalonia, Spain; 5 National Genotyping Center (CeGen) Barcelona Genotyping Node, Center for Genomic Regulation (CRG-UPF), Barcelona, Catalonia, Spain; Max Planck Institute for Evolutionary Anthropology, Germany

## Abstract

**Background:**

Understanding the genetic contribution to phenotype variation of human groups is necessary to elucidate differences in disease predisposition and response to pharmaceutical treatments in different human populations.

**Methodology/Principal Findings:**

We have investigated the genome-wide profile of structural variation on pooled samples from the three populations studied in the HapMap project by comparative genome hybridization (CGH) in different array platforms. We have identified and experimentally validated 33 genomic loci that show significant copy number differences from one population to the other. Interestingly, we found an enrichment of genes related to environment adaptation (immune response, lipid metabolism and extracellular space) within these regions and the study of expression data revealed that more than half of the copy number variants (CNVs) translate into gene-expression differences among populations, suggesting that they could have functional consequences. In addition, the identification of single nucleotide polymorphisms (SNPs) that are in linkage disequilibrium with the copy number alleles allowed us to detect evidences of population differentiation and recent selection at the nucleotide variation level.

**Conclusions:**

Overall, our results provide a comprehensive view of relevant copy number changes that might play a role in phenotypic differences among major human populations, and generate a list of interesting candidates for future studies.

## Introduction

Although any two humans are 99.9% identical at the nucleotide sequence level [1], [2], many phenotypic differences are apparent in individuals within the same and from distinct human populations. Genetic diversity underlying the remaining 0.1% nucleotide differences has been postulated to contribute to phenotypic diversity among humans, and to population-specific susceptibility to disease and variability in the response to pharmacological treatments [3]–[5]. The different prevalence of Mendelian diseases reflects variability in allele frequencies for specific genes and haplotypes, and the relevance of ethnic background in the susceptibility to disease is recognized for several disorders [6], including cystic fibrosis [7]–[9], sickle cell anemia [10]–[14], and deafness [15], among many others. Similarly, there are differences in the prevalence of common disorders and associated genetic variants in human populations, such as the factor V Leiden (venous thromboembolic disease [16], [17]), variants in the *CARD15* gene (Crohn's disease [18], [19]), the CCR5–Δ32 variant (human immunodeficiency virus (HIV) infection and progression [20]), and *APOE* e4 (Alzheimer's disease [21]). In addition, population-specific genetic variants could also influence the metabolism and response to drugs, exemplified by the population variability in the acetylating activity of the N-acetyltransferase 2 *(NAT2)* gene [22]. However, despite the progress on the identification of genes for inflammatory bowel disease and neurodegenerative disorders, little is known about the genetic contribution to the prevalence differences of other complex traits among human groups [6].

The current knowledge of divergence among major populations and subpopulations is based in the study of different sources of genetic variation within and amongst human populations [23]–[26]. The analysis of nucleotide and structural variability on human populations has shown that genetic clusters closely correspond with human groups defined by ethnicity or continental ancestry [27]–[29]. In agreement with human African origins, a higher amount of SNP allelic variability has been found in individuals of African ancestry, while individuals of Asian origin show the smallest amount of nucleotide diversity [27]. Another major conclusion from these studies is that for most nucleotide and small genomic changes (in/dels), intra-population differences among individuals account for the majority of variation, while differences among major population groups represents a minimal fraction [26], [29]–[31]. However, this variation cannot be neglected.

Despite that the existence of structural variation in the human genome has been known for a while (see [32] and [33] for review), we only recently have started to recognize its real scope and wide-spread nature [34]–[37]. This variation is present in different forms, including insertions, deletions and inversions. Due to the relative ease of identification, copy number variants (CNVs) are the most studied and best recognized form of structural variation, and have attracted much attention because of its potential to affect gene expression [38], [39].

Most studies aiming at the discovery of CNV regions have made use of genome-wide approaches based on different array-based comparative genomic hybridisation (aCGH) and comparative genomic intensity (aCGI) platforms, and have provided partial evidences of their nature, real size and/or population distribution. The first comprehensive map of CNVs was produced in late 2006 [35] by the analysis of 270 HapMap reference samples using high-density BAC arrays and Affymetrix 500 K EA chips. It allowed the identification of 1,447 CNV regions (∼360 Mb; 12% of human genome), showing a 43% concordance between the two platforms. A subsequent analysis of 71 individuals from three different human populations using an equivalent high-density BAC array revealed 315 CNV regions, 40% of them not having been previously reported [40]. A targeted high-density analysis of 1,153 known CNVs (using oligonucleotide-based aCGH at a 1 Kb resolution) on 30 HapMap samples, showed that 76% of CNV regions were at least 50% smaller than previously reported [41]. More recently, using a dense aCGI platform (Affymetrix 6.0), McCarroll and co-workers refined the CNV knowledge on the HapMap samples and identified 3,048 CNV regions, 40% of them not overlapping with previously reported variants [42]. Furthermore, an in-depth study using the Illumina HumanHap550 BeadChip surveyed copy number variation on individuals from 29 different human worldwide populations [29]. Although little experimental validation was performed, their analysis of 396 non-unique autosomal loci revealed that CNV distribution parallels the population structure reported by SNP data. All these studies highlight that, except for those focusing on single or a reduced number of loci [43]–[45], more detailed analysis of the loci is necessary to determine their potential contribution to normal phenotypic variation and disease susceptibility. In addition, although a big proportion of the human genome has been suggested to be copy-number variable, little is known about the functional consequences of differences in population distribution of CNVs.

A series of studies have already linked rare and common CNVs to complex diseases (such as Alzheimer's disease, HIV and AIDS susceptibility, chronic pancreatitis, autism or schizophrenia) (reviewed in [46]) and established etiopathogenesis connecting changes in copy number with alteration of expression levels of dosage-sensitive genes. Apart from the direct effect on gene expression, a link between some structural variants and predisposition to disease-causing rearrangements has been proven, especially for genomic disorders [47]–[50]. Thus, population-specific differences of CNV frequency could make a certain human population more prone to suffer certain types of disorders.

In this study we report the identification of CNVs that are present in very different frequency in individuals from the three phase I/II HapMap analysis panels [27] (YRI - Yoruba in Ibadan, Nigeria; CEU - Utah residents with ancestry from Northern and Western Europe; and CHB+JPT - Han Chinese in Beijing, China and Japanese in Tokyo, Japan), representatives of sub-Saharan Africa, Europe and East Asia, respectively. Most of the genomic variants detected have already been described as CNVs, involve genes, and modify, to a certain extent, their expression levels. Furthermore, we show that these CNVs are sufficient to discriminate population structure at the continental level, and that some of them are embedded in population-specific haplotypes. We finally provide evidences of the effect of natural selection on the variants identified.

## Results

### Identification of population differences in CNV regions

**Figure 1:**
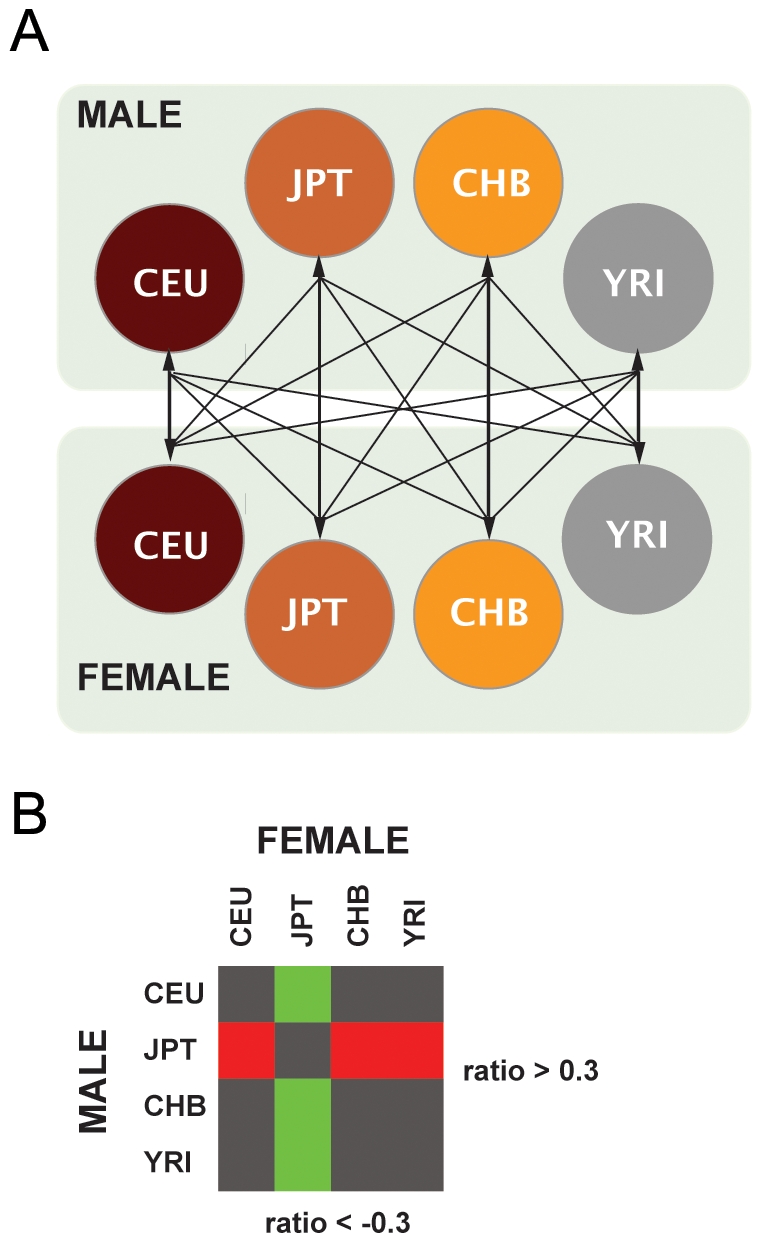
General Strategy Used in the Analysis of Copy Number Variants (CNVs) with Population-Specific frequency changes. (A) Pools of genomic DNA of 25 unrelated males and 25 females from the four HapMap populations were prepared and hybridized to the different array platforms. In total, sixteen inter-population CGH experiments were performed in each platform. The circular design of the hybridization pairs (panel A) allowed us to assign the most likely population to carry the variation, assuming the most parsimonious scenario. (B) This combination of hybridizations also allowed us to minimize the calling of CNVs on spurious positive signals. For instance, for a CNV to be called as a YRI-specific loss, the hybridizations of CEU, CHB and JPT male pools were required to show an increased hybridization signal with respect to the YRI female pool (log_2_ ratio≥0.3, green squares), the hybridization of the YRI male pool versus the rest of pools had to show decreased signals (log_2_ ratio≤−0.3, red squares), and the rest of combinations should show values around 0 and not above the |0.3| threshold (dark squares).

We have used aCGH [51], [52] on Agilent and BAC-based platforms to identify CNVs with different frequencies in three representative human populations of African (YRI – Yoruba in Ibadan, Nigeria), Asian (JPT – Japanse in Tokyo, Japan, and CHB – Han Chinese in Beijing, China), and European (CEU – Utah residents with ancestry from Northern and Western Europe) ancestry. In order to dilute inter-individual variation and to enrich inter-population differences, we pooled 50 unrelated DNA samples from each of the four human groups studied in the HapMap project [5], [28]. For each group, we set up independent pools of males and females, and carried out a total of sixteen aCGH hybridization experiments on each platform, by confronting sex-unmatched pools of individuals from the different populations (Figure 1A). As a control, we performed an intra-population male *versus* female hybridization to discard the probes that called a copy number difference in this situation (see Methods), since we do not expect copy number variability between genders of the same group, other than those affecting the sexual chromosomes. The circular design of the hybridization experiments (Figure 1A) allowed us to determine the most likely population that carries the variant (increase or decrease in copy number) with respect to the other populations, assuming the most parsimonious scenario. In addition, this design also allowed us to minimize the presence of spurious positive signals (Figure 1B).

The global analysis of the aCGH data on the three array platforms yielded a total of 111 loci whose copy number state frequencies differed among populations (Table S1), including 59 gains, 43 losses, and 9 complex changes which showed a range of variation that we could not assign to a single population (see Methods). Because of the similarities observed between the JPT and CHB groups, and since they both belong to a population of Asian ancestry, we grouped them and will use the ASN (Asian) term to refer to them. From the copy number changes that could be assigned to a population, 26 were CEU-specific, 23 ASN-specific and 53 YRI-specific (Table 1). The amount of copy number changes found in the YRI population doubles that of any of the other individual populations, and is concordant with previous observations of higher frequency of changes for small, intermediate and large-scale variants and insertion/deletion polymorphisms [35], [53]. Of the identified regions, 61% (n = 68) overlap with previously reported CNVs, and 32% (n = 35) overlap with segmental duplications.

**Table 1:** Combined results of Copy Number Variant (CNV) changes in HapMap populations using genome-wide aCGH platforms.

	Gain	Loss	Complex	Total
TOTAL	59	43	9	111
CEU	13	13	0	26
ASN	10	13	0	23
YRI	36	17	0	53

Number of CNV calls inferred from log_2_ ratio data from the set of array comparative genome hybridizations performed. The type of variation and population-specificity is assigned assuming the most parsimonious scenario (see Methods).

### Validation of the aCGH findings

To confirm the changes detected with the aCGH platforms, we performed exhaustive validation experiments using Multiplex Ligation-dependent Probe Amplification (MLPA) [54] on individual DNAs from the HapMap collection. Depending on the length of the variable segment, one or two MLPA probes per region were designed, preferentially within genes (Table S2). We split the predicted CNVs into three different categories depending on the number of consecutive BAC or oligonucleotide probes that supported the altered segment (1, 2 or >2 probes per region, respectively; Table 2). We decided to validate putative CNVs from all three groups, although we anticipated that the lower the number of array probes supporting each segment the higher the likeliness of being a false positive. In total, we analyzed 152 probes in 4 different probe mixes (see Methods) that interrogated 93 of the 111 CNV regions, including 60 CNVs already described in the literature and 33 potentially novel CNVs. Overall, after validation, 47% of the tested loci (n = 44) turned out to be variable, and the majority of them (86%; n = 38) corresponded to already known CNVs, thus only 6 were novel. Out of the 44 variable regions, 33 (75%) were concordant with the results observed in the aCGH experiments, while the remaining 11 did not coincide with the aCGH prediction or did not show significant differences among populations. As expected, the validation rate of the CNVs varied considerably depending on the number of supporting array probes, and was low (33%) for group 1 (CNVs composed of only 1 altered probe) and much higher for groups 2 and 3 (73% and 72%, respectively) (Table 2). On the other hand, our results are consistent with the observations made by others [36], [41] in the sense that the boundaries of many variants reported so far are not precisely defined, because different MLPA probes located within the same theoretical CNV did not behave uniformly (Figure S1).

**Table 2:** Validation of CNV changes identified in HapMap populations using different aCGH platforms.

# Probes	Combined			BACs		Oligos	
	Validated n (%)	Not validated n (%)	Total	Validated n (%)	Not Validated n (%)	Validated n (%)	Not Validated n (%)
1	20 (33)	40 (66)	60	11 (73)	4 (27)	9 (13)	58 (87)
2	11 (73)	4 (27)	15	5 (56)	4 (44)	6 (100)	0 (0)
≥3	13 (72)	5 (28)	18	4 (80)	1 (20)	13 (93)	1 (7)
Total	44 (47)	49 (53)	93	20 (69)	9 (31)	28 (32)	59 (68)

Combined and platform-specific validation success of varible regions identified by aCGH. **BACs**: BAC-based array-CGH platform. **Oligos**: oligonucleotide-based array-CGH platform.

Although direct comparison is difficult, as CNV region boundaries identified by arrays cannot be well defined and different array platforms use probes located in different regions, we have estimated to what extent our results matched with previous data deposited in the database of genomic variants (DGV - [55]) by evaluating reported frequencies of overlapping CNVs (Table S3). Of the 33 CNV loci validated as showing populations differences, in 13 of them we could not establish a concordance with published results, either because the CNV was not previously reported, there was not enough population information to compare, or there were inconsistencies in the available data. In 80% of the remaining 20 cases (16/20), the copy number frequencies we obtained matched with the available population data; while in the other 4 cases the information we obtained did not agree with what it had been published beforehand. Thus, most of the CNVs with population differences are independently confirmed by various methods and supported by previously existing evidences.

**Figure 2:**
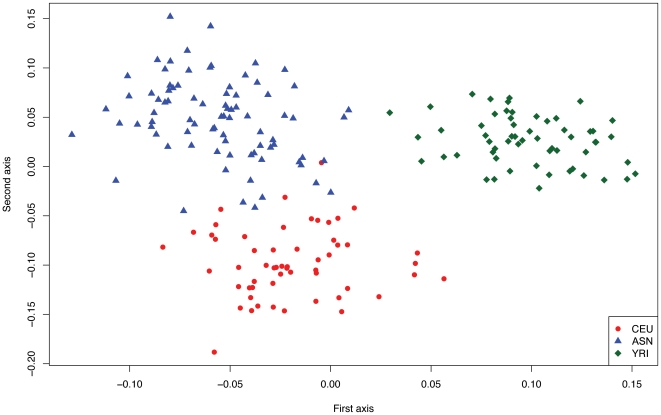
Principal component analysis of MLPA data in HapMap populations. We decomposed the genetic variation within MLPA-estimated copy number into the first two principal axes [56], plotted each data point, and colored them according to the population of origin. Thirty percent of the total variance is explained by the two first principal components (PC1 16,62%, and PC2 13,38%), and it is evident that the first principal component discriminates variation inherent to the population of African ancestry (YRI), while the second component differentiates the populations of European (CEU) and Asian (ASN) ancestries.

To test for the existence of population stratification based on CNVs, we took advantage of the MLPA data generated for all analyzed loci to perform an *a priori* principal component analysis of the copy number information estimated for each individual [56]. We estimated that thirty percent of total variance is explained by the two principal components (PC1 16,62%, and PC2 13,38%) and we observed that using these two axes the majority of individuals consistently cluster according to their population of origin, thus supporting the idea that the three human groups are characterized by a markedly different composition of the identified CNV regions (Figure 2).

### Gene content in CNV regions showing population differences

Thirty of the 33 CNVs that we validated to show population differences contain known coding genes, totaling 40 RefSeq genes (Table S1). The three remaining CNV regions are completely devoid of coding sequences, but using the Evolutionary Conservation of Genomes Browser [57] we identified conserved segments that might have some potential biological function that has not yet been uncovered (data not shown).

To characterize the nature of the genes located within the set of validated CNVs we carried out a GO-term analysis using the Gene Ontology Tree Machine [58]. This analysis revealed a significant enrichment of genes involved in immune response, which is consistent with previous studies [59]–[62]. Other functional categories enriched when compared both to the rest of human genes or the set of genes located within known CNVs (Tables S4 and S5) were protein and lipid metabolism genes, and genes related to the extracellular space. Therefore, the genes identified are mainly related to interaction and adaptation to the environment. In addition, we performed an analysis of the rate of non-synonymous (Ka) to synonymous (Ks) nucleotide changes (Ka/Ks) between humans and chimpanzees to test whether the genes with changes in copy number among human populations are characterized by a different selective regime in the ‘recent’ evolutionary history, which could be consistent with a role in environment response. We found that the average Ka/Ks ratio of our set of genes is not significantly different from what it would be expected by chance compared to the rest of coding genes in the genome. Similar results were obtained using Ka/Ks estimates retrieved from Ensembl (Tables S6 and S7). Therefore, we conclude that the CNV gene set has been subjected to analogous functional constraints than other genes during hominoid evolution.

Several of the genes coinciding with the CNVs detected here attracted our attention because of their biomedical relevance and potential involvement in human adaptation to environmental pressures. For example, the Haptoglobin-related protein (HPR) is found in a CNV region that exhibits a higher copy number in the YRI individuals than in the individuals from the other two populations analyzed, and could be related to adaptation to sleeping sickness disease (see Discussion). Other interesting genes located within regions with considerable copy number differences among populations include several that were previously known to be variable and associated to different biomedical conditions (*GSTT1*, *UGT2B17*, *PRSS2* and *APOBEC*), as well as others that, although associated to disease phenotypes, have not been reported to be variable among populations. Among those, there are several promising candidates that might be influencing different population prevalence of diseases like age-related macular degeneration (*CFHR3*) [63]–[67], cardiovascular disease (*LPA*) [68]–[73] and chronic obstructive pulmonary disease (*HCK*) [74].

### Contribution of the CNV changes identified to gene expression variability

The most intuitive functional consequence of a change in genomic dose is a change in gene-expression levels (i.e. more copies imply higher expression, and vice versa). To check this, we identified a total of 23 RefSeq genes contained within the 44 validated CNV regions (regardless of showing population differentiation) for which expression levels in lymphoblastoid cell lines, derived from the same samples we analyzed, had previously been reported [38]. Of those, 16 transcripts exhibited average expression levels above the threshold of the negative controls and were further considered. First, we wanted to know to what extent global expression level of genes within the variable regions could be attributable to copy number differences. In total, five transcripts showed significant correlation (*P*<0.0031, after Bonferroni correction) between copy number and expression levels for the three populations combined, which represents a slightly higher proportion (31%) than previously reported [38] (Table 3). Second, when considering only the CNVs with population-specific frequency changes, 10 out of 14 (71%) transcripts showed significant expression differences among populations (Kruskal-Wallis Test; *P*<0.0036; after Bonferroni correction; Table 3) and we could assess a positive correlation between expression levels and the copy number in 60% of them (6 out of 10) (Figure S2). One interesting instance is the *UGT2B17* gene, whose expression levels have been previously associated with specific cis-acting SNPs [39], [75], that we have found to be tightly correlated with the copy number of the gene (Table 3), as previously reported by others [38], [76]. Thus, it seems reasonable to postulate that the differences in copy number are responsible of controlling expression, and it is possible that the SNPs previously identified are mere tags for the different copy number alleles. Nevertheless, when assessing the relationship between CNVs and gene-expression, we have to keep in mind that we solely used expression data that was collected from a single tissue. In addition, it is important to note that other genes were contained within the identified CNVs, but were not included in our analysis because their expression levels were not reported in the above-mentioned study or because the MLPA probes we used did not coincide exactly with the gene. Given the poor definition of CNV boundaries reported in the currently available databases observed by others [36], [41] and us, we decided not to take into account the genes for which we have not directly inspected copy number.

**Table 3:** Expression differences in lymphoblastoid cell lines from HapMap samples and correlation with copy number at CNV loci.

CNVID	MLPA probe	RefSeq	Gene ID	Illumina Probe	CEU Mean	CEUSD	ASN Mean	ASN SD	YRI Mean	YRI SD	Adj. *r^2^*	Kruskal Wallis
71	Chr13_Pop_1	NM_198441	-	GI_38348197.S	6,194	0,078	6,183	0,07	6,216	0,069	0,046 *	7,673
78	Chr16_Pop_1	NM_015027	PDXDC1	GI_39930344.S	6,244	0,114	6,181	0,079	6,164	0,092	0,001	20,038 *
83	Chr16_70653608	NM_020995	HPR	GI_45580722.S	6,15	0,064	6,145	0,071	6,187	0,049	0,019	19,369 *
88	chr17_335_A	NM_032258	TBC1D3	GI_14149984.S	6,929	0,202	7,052	0,188	7,116	0,382	0,012	17,203 *
89	chr17_415_A	NM_015443	KIAA1267	GI_41152088.S	8,9	0,155	9,025	0,222	9,124	0,175	0,024	36,522 *
90†	chr17_420_A	NM_006178	NSF	GI_11079227.S	10,818	0,311	10,372	0,303	10,45	0,273	0	57,14 *
90†	chr17_420_B	NM_006178	NSF	GI_11079227.S	10,818	0,311	10,372	0,303	10,45	0,273	0,002	57,14 *
91	chr17_429_A2	NM_006310	NPEPPS	GI_15451906.S	9,551	0,236	9,576	0,27	9,568	0,154	0	0,636
91	chr17_42973027_43038163_A	NM_006310	NPEPPS	GI_15451906.S	9,551	0,236	9,576	0,27	9,568	0,154	0,01	0,636
91	chr17_429_B2	NM_006310	NPEPPS	GI_15451906.S	9,551	0,236	9,576	0,27	9,568	0,154	0,001	0,636
98	A_14_P112851	NM_004283	RAB3D	GI_18677727.S	6,568	0,141	6,559	0,111	6,517	0,112	0,008	5,231
11	A_14_P114658	NM_015690	STK36	GI_34222107.S	8,559	0,373	8,499	0,313	8,581	0,251	0,029	3,861
103	A_14_P105195	NM_002110	HCK	GI_30795228.S	7,641	1,011	7,062	0,683	7,293	0,839	0	11,528 *
111	Chr22_22690592	NM_000853	GSTT1	GI_4504184.S	7,786	0,986	7,147	1,154	7,456	1,237	0,889 *	10,591 *
110	Chr22_Pop_2	NM_004900	APOBEC3B	GI_22907024.S	7,962	0,573	7,656	0,944	8,242	0,396	0,758 *	17,428 *
25	Chr4_69231671	NM_001077	UGT2B17	GI_4507820.S	9,241	1,361	6,789	1,478	9,226	1,044	0,942 *	80,664 *
33†	chr5_130_B_LYRM7	NM_181705	LYRM7	GI_32171235.S	8,045	0,324	8,152	0,284	7,983	0,235	0	12,368 *
37	Chr6_Pop_1	NM_005577	LPA	GI_5031884.S	6,191	0,131	6,118	0,076	6,146	0,075	0,001	13,544 *
38	Chr6_32594054	NM_002125	HLA-DRB5	GI_26665892.S	13,107	1,05	11,939	2,591	9,94	2,954	0,681 *	29,487 *

Mean and standard deviation (sd) of expression levels were calculated from unrelated individuals belonging to each population. To test correlation of copy number and expression levels we used an ANOVA and fitted a linear model using the two variables. The Kruskal Wallis test was calculated to assess differences in expression levels among populations.

†Denotes that these CNV regions do not show population differences. **CNVID**: CNV Identifier. *P* values with an asterisk (*) denote that it is significant after Bonferroni correction.

### LD and SNP haplotype structure of population variable CNV regions

Although only a small proportion of human copy number variation can be attributed to population differences [29], [35], in this work we specifically sought to enrich for this type of changes. In order to check in more detail the underlying genetic architecture of these regions, we examined the nucleotide variation data from the HapMap project.

**Figure 3:**
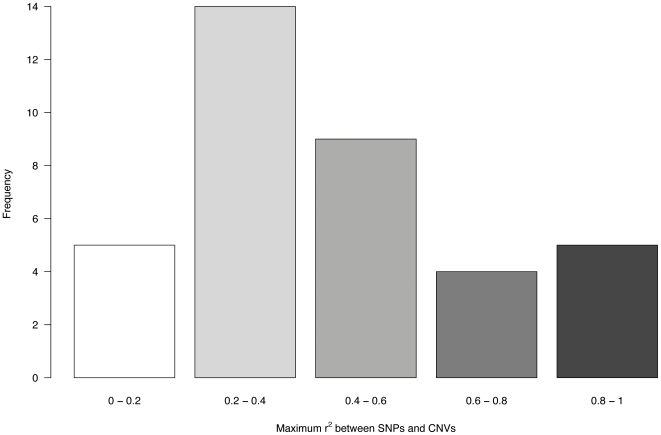
Linkage disequilibrium between HapMap SNPs and CNVs. The distribution of maximum measured *r^2^* value for the SNPs analyzed for each CNV is plotted in the histogram, which represents the frequency of findings (Y axis) in each range of LD values (X-axis).

Initially, we used the available HapMap genotype information of each individual to search for evidences of LD between the identified CNVs and flanking SNPs. The median of the maximum *r^2^* values for single SNPs located up to 100 kb around the boundaries of each CNV was 0.37, indicating a low overall disequilibrium between SNPs and copy number alleles. Nevertheless, very heterogeneous disequilibrium patterns were observed for individual CNVs. Four loci show very high LD (*r^2^*>0.8) with surrounding SNPs, and others present low levels of disequilibrium (Figure 3). This indicates that, as reported previously [35], [76]–[78], at least in some cases it might be feasible to define tag-SNPs as proxies for CNV alleles in certain populations that could be helpful in future analyses of those variants (Table S8). Given the complexity of variable regions and the difficulty to genotype within and around them, it is worth noting that the average number of SNPs that could be analyzed (1/2059 bp) was slightly lower than the average density in HapMap (1/1495 bp; according to the total number of polymorphic common-to-all-population SNPs) [28].

Due to the population specificity of our set of CNVs, we expected to find some of them embedded in particular haplotypes, and therefore to be able to identify different patterns of LD between surrounding SNPs and copy number alleles in each population. When possible, we performed pairwise comparisons of LD patterns between populations using the Spearman rank correlation value (ρ), and found that they are quite different in most cases (Table S9). Thus, the regions explored here do not seem to follow the general rule of preserved LD patterns across populations that are observed globally in the genome [27], [28], [79], and this is a potential evidence of recent selection. In extreme cases, in which the CNV appears exclusively in a single population, the test could not even be performed. On the other hand, we also identified three cases in which there is a high correlation value. This observation is consistent with a high degree of concordance in the LD patterns observed in the different populations (i.e. CNVs interrogated by probes chr5_150_A, A_14_P133280 and Chr1B_pop_2), and likely indicates that these CNVs appeared as a single event predating the out-of-Africa human migrations.

To examine more carefully the degree of population differentiation at the SNPs that are in highest LD with the identified CNVs (tag-SNPs) we used the *F_ST_* measure [80], [81]. *F_ST_* values range from 0 (no differentiation) to 1 (complete differentiation) and can provide a measure of the action of natural selection on these regions. It is known that negative and balancing selection tends to decrease *F_ST_* in a locus-specific manner, whereas local positive selection tends to increase *F_ST_* [82], although unusual *F_ST_* values are not a definitive proof of the action of selection. According to the distribution of the available *F_ST_* data for HapMap SNPs [83], [84], we considered values above 0.20 (corresponding to the top 15–20^th^ percentile of the distribution of *F_ST_* values between each pair of populations) as evidence of population differentiation. Ten of the tag-SNPs had global (i.e. across the three populations) *F_ST_* values above this threshold. In seven out of the ten cases, the most differentiated population coincided with the one in which the population variable CNV was identified (Table S10). These results strengthen our initial findings with the aCGH and MLPA experiments, and indicate that the CNV differences among populations are often replicated in linked SNPs.

Finally, the analysis of haplotype structure can provide a good estimate of the contribution of positive selection in our regions of interest, which might account for the observed population differences [85]. To test for recent selection events, we used the available Integrated Haplotype Score (*iHS*) data, which takes into account different measures of haplotype length and composition in each population [86]. We found that seven tag-SNPs showed signals of recent positive selection (|*iHS*|≥2); and in six of those, the extreme *iHS* values were corresponded to the population in which we observed the change in CNV frequency (Table S10). Therefore, there are evidences that, at least in some cases, the change in frequency of a CNV in a population was associated to positive selection acting on itself or on a linked nucleotide variant.

## Discussion

Given the unexpected extent of structural variation among individuals, it is very likely that there is an important contribution of such variants to phenotypic differences, including predisposition to common and complex disorders. Although different authors have reported the existence of CNVs in individuals from different geographical locations [29], [35], [40], [45], experimental validation is lacking for a number of the reported variants. To uncover the main part of such variability, we have performed whole genome array-based comparative genome hybridization of pooled DNA from different populations, and made an important effort to provide validation of the most relevant findings. In addition, we have combined different available sources of information to estimate the functional and evolutionary relevance of these changes.

Given the resolution and coverage of our arrays, we have been able to identify a “relatively” small set of CNVs that show significant differences in population distribution. Despite the stringent criteria for CNV calling, an important number of them could not be validated by MLPA analysis, especially those supported by just one array probe, and several reasons can account for that. First, it is possible that some of the aCGH observations were indeed false positives due to the large number of regions assayed in the arrays (i.e. the larger the number of tests, the larger the type I error). Second, in the case of the variable loci reported by a single probe in the oligonucleotide arrays (Table 2), it was not always possible to design the MLPA probes in the exact same place, and they were designed within one kb around the array probe. As a result, it is possible that the probe ended up located outside of the really variable region. Finally, it is also possible that some of the loci reported had a basal copy number that is too high to be correctly measured by MLPA, that is known to reliably distinguish copy numbers between zero and five copies [43], [45], but its sensitivity greatly decreases when higher copy numbers are measured. This is likely the case of the chemokine *CCL3L1* gene that appeared as variable in the array but could not be confirmed by MLPA, although we know that it is indeed highly variable, with median values of 3 and 6 copies per diploid genome in non-Africans and Africans, respectively [87]. On the other hand, despite the limitations of matching differently defined CNVs, the comparison of the MLPA validation data with previously reported information has revealed a very good concordance, with 80% of the CNVs we identified agreeing with previously published data. It is a fact that genome-wide platforms have been a great tool for obtaining a global view of copy number variation, but big efforts of validation on individual loci will be required in the future prior to establish the impact of individual CNVs in human health. In this regard, MLPA has proven to be an easy, efficient, reliable and inexpensive method to validate copy number variants in large sets of samples.

CNVs have been suggested to play an important effect on gene-expression levels. In our study, 60% (6 out of 10) of the genes showing inter-population expression differences associate with copy number changes, which reinforce the view that these variants are indeed important modulators of expression. The list of genes whose expression differs among human groups and is correlated to copy number includes, amongst others, genes involved in xenobiotic detoxification (*GSTT1*), cellular immunity (*APOBEC3B* and *HLA-DRB5*) and hormone metabolism (*UGT2B17*) (Table 3 and Table S1). Although in some cases we have been able to corroborate the intuitive higher dose – higher expression hypothesis, the effect of CNVs on expression can be far more complex. Depending on the modification of the regulatory landscape of specific genes, it has been shown that CNVs can affect distant transcripts in *cis* or in *trans*, and even result in a negative correlation between expression and copy number (more copies, less expression) [38]. In addition, the inherent complexity of the regulation of gene expression, with temporal and tissue-specific patterns, makes it difficult to evaluate the global effect of structural variation on expression from the available data coming from only one tissue. In our case, the total number of genes whose expression levels could be evaluated (i.e. because they had levels above the negative control threshold) in relation to copy number was only half of the total number of genes contained within the regions identified.

Despite these limitations, we have observed a number of population-specific copy number variants that affect genes associated to different phenotypes and disorders, and are very interesting candidates for future studies. For instance, we found that YRI have significantly lower copy number of the *CFHR3* gene (Complement Factor H-Related gene 3) than CEU and ASN individuals. In addition, we have observed that this variant is well tagged by a SNP (rs16840639; *r^2^* = 0.79) that shows a significant population differentiation and exhibits signatures of recent positive selection. Interestingly, the incidence of age-related macular degeneration (AMD) shows racial differences [88], [89], with lower prevalence among individuals of African origin. It is thus tempting to speculate about a connection between an AMD protective role of the deletion [63]–[67], the population-specific pattern of AMD prevalence, and our findings about copy number population differences. Another attractive candidate is the haptoglobin-related protein gene (*HPR*), which is found in a CNV that exhibits a higher copy number in Yorubans than in the other two populations analyzed, and the linked SNPs also show high *F_ST_* values among populations. The HPR is a key component of the serological innate immunity system that protects humans and other primates against *Trypanosoma brucei brucei* infection [90]–[92], which causes sleeping sickness disease [93], [94]. Therefore, it seems likely that the limited geographical distribution of the *Trypanosoma brucei*'s vector, the TseTse fly, which is endemic to Africa [95], has influenced the elevated frequency of the high copy number alleles in individuals of the African population. Finally, elevated plasma levels of *LPA* gene product (lipoprotein A or Lp(a)) are associated with increased risk for atherosclerosis and its main manifestations: myocardial infarction, stroke and restenosis [21], [68]–[71]. In this direction, the increase in copy number of the *LPA* gene observed in the ASN population is associated with lower gene-expression levels (Table 3), which might explain, at least in part, the reduced risk of cardiovascular disease in individuals of this background.

Genes contained in CNV regions with population copy-number state frequency differences are enriched in functions related to interaction with the environment. For that reason, the different frequencies of these variants could be a sign of recent local adaptation events that have favored certain haplotypes over others in response to specific environmental conditions, related for example to dietary restriction or exposition to pathogens. By looking at the associated nucleotide variation, we have tried to discern if the differences could be attributable to some biological adaptation process driven by natural selection or just to random genetic drift. The diverse LD patterns among populations, the extensive population differentiation (high *F_ST_*) and the presence of long haplotypes (high absolute values of *iHS*) point to recent positive selection acting to increase CNV frequency in a particular population. Nevertheless, in other cases, the available evidences suggest that genetic drift is the most likely responsible for the observed differences in CNV population frequency. Therefore, taken as a whole, the data obtained from the tag SNPs points that there is not a general pattern of selection for the different CNVs herein identified.

Our results highlight the importance of CNV differences among populations and, although more detailed studies would be needed to characterize their real functional consequences, provide a validated set of CNVs that might contribute to phenotypic and disease susceptibility differences among human populations. The identification of these variants emphasize the utility of current available platforms to evaluate copy-number differences genome-wide and pinpoint the need to develop specific technologies to reliably genotype CNVs in multiple individuals, since currently available array platforms and other proposed approaches (CNV-SNP tagging) might be hampered by the significant differences in population distribution of some CNVs. The work presented here represents a first step towards characterization of population differences in genome structure and will need to be revisited when denser and more precise tools to study structural variation are available. In addition, it would also be important to evaluate the impact on population-specific variation of balanced rearrangements not detected by aCGH, such as inversions, that, although putatively less frequent, could have a total genome coverage and potential contribution to variation at least as important as that of copy number changes [36], [37].

## Materials and Methods

### aCGH on pooled DNA samples from different populations

We performed array-based Comparative Genome Hybridization (aCGH) on pools of samples from different populations to detect CNVs. To obtain the pools, we used DNA extracted from lymphoblastoid cell lines from the HapMap collection (obtained from the Coriell Institute for Medical Research). The samples consisted of 50 unrelated individuals (25 males and 25 females) from four population groups: Yoruba from Ibadan, Nigeria (YRI), Caucasians of European descent from Utah, USA (CEU), Japanese from Tokyo, Japan (JPT), and Han Chinese from Beijing, China (CHB). In order to complete the 50 unrelated samples from Asian ancestry, we included individuals from HapMap Plate 05. Individual DNA quality was assessed by alkaline gel electrophoresis and the concentration was measured using the PicoGreen® assay (Invitrogen, Life technologies, Carlsbad, CA, US). 500 ng of DNA from each individual were combined to obtain each population-specific pool, which gave enough DNA for the several experiments using different array platforms.

Three different genome-wide platforms were used to perform the CNV detection: an in-house developed BAC-based array consisting of ∼32.000 BAC-derived probes from the Human 32 K clone set library (kindly provided by Dr. Joris Veltman), a gene-centered oligonucleotide array of ∼44.000 probes (Human Agilent 44 K; Agilent Technologies Inc, Santa Clara, CA, US) and a genome-wide oligonucleotide array of ∼185.000 probes (Human Agilent 185 K). The hybridization, washing and scanning of the BAC-arrays was performed as described elsewhere [96], with minor modifications. Briefly, 400 ng of test and control DNA were labelled by random priming using the BioPrime Array CGH Genomic Labelling System (Invitrogen, Life technologies, Carlsbad, CA, US). The hybridization, washing and scanning of Agilent arrays was performed following manufacturer's instructions with minor modifications. For each array, each population-specific pool was hybridized against all the rest population pools in a sex-unmatched specific manner (i.e. the male pool from a certain population is hybridized with the pools of the opposite sex of the other populations - Figure 1A). In addition, we performed a sex-unmatched, intra-population hybridization to discard clone and oligonucleotide probes behaving abnormally. Arrays were scanned using an Agilent G2565BA MicroArray Scanner System (Agilent Technologies Inc., Santa Clara, CA, US) and the acquired images were analyzed using GenePix Pro 6.0 software (Axon, Molecular Devices, Toronto, Canada) using the irregular feature finding option. Extracted raw data was filtered and Lowess normalized using Bacanal [97], an in-house web server implementation of the Limma package developed within the Bioconductor project [98].

Genomic imbalances were determined based on the average log_2_ of the Cy5/Cy3 ratios of the spotted replicates, and regions were considered as amplified or deleted when probes exceeded the ±0.3 threshold. The design of the hybridizations allowed us to establish *a priori* combinations of hybridization intensity patterns to assign the most likely population to carry the variation, assuming the most parsimonious scenario (Figure 1B). The combination of hybridizations also allowed us to minimize the calling of CNVs on spurious positive signals. For instance, for a CNV to be called as a YRI-specific loss, the hybridizations of CEU, CHB and JPT male pools were required to show an increased hybridization signal with respect to the YRI female pool (log_2_ ratio≥0.3, green squares), the hybridization of the YRI male pool versus the rest of pools had to report decreased signals (log_2_ ratio≤−0.3, red squares), and the rest of combinations should reveal equal intensities (dark squares) (Figure 1B). Any number of consecutive probes that exhibited log_2_ ratios consistent with a population-specific CNV call after the analysis of the combination of hybridizations on the same platform was considered for further analysis. We discarded probes/clones that exhibited discordant log_2_ ratios among the different combinations. The high degree of similarity between the patterns obtained in the CHB and JPT samples prompted us to combine the data from both populations and to treat the two populations as a single one named ASN (for Asian). Data obtained from each of the three platforms was considered and analyzed independently.

### Detection limits of aCGH

In order to estimate the lower limit of aCGH to detect copy number changes among samples, we set up an experiment in which we diluted DNA from an individual carrying a deletion of known size at 7q11.23 (typical Williams-Beuren Syndrome deletion) into a pool of normal DNA. We determined that we were able to call a copy number change when the alteration was present in between 20% and 30% of the chromosomes that participated in the hybridization (data not shown).

### MLPA validation and data analysis

Oligonucleotides for each probe were designed following the recommendations of the proprietary of the MLPA technology (MRC-Holland) using the automated web application called MLPA ProSeek [99]. A total of 133 locus-specific MLPA probes were combined in 5 different mixes to validate the findings. Each probe mix was composed of about 30 probes targeting selected genomic loci (Table S2). In the case of CNV loci reported by a single oligonucleotide probe in the array, the MLPA probe was designed within a window of 1 kb around the original position; otherwise, it was designed within the altered region. Each probe mix contained two extra control probes interrogating regions that are deleted and duplicated in the positive control samples that were included in each experiment, but are invariable in normal individuals. For validation, we analyzed all individual samples included in the pools plus the rest of individuals in HapMap plates 01 (90 CEU), 02 (90 ASN), 03 (90 YRI) and 05 (90 ASN) (Coriell). In addition, control samples were included in each plate: two DNAs with known aberrations (10q duplication, 22q deletion) plus 4 replicates of each of the male pools used in the hybridizations. MLPA was performed as described elsewhere [54], with minor modifications.

Although DNA concentration was controlled during the preparation of the plates, for data analysis each sample was normalized internally by dividing the height of each individual peak by total sum of peaks heights (dosage quotient). In order to compare results among plates, individual peak dosage quotients of each sample were normalized against the mean dosage quotients of the control samples included in each plate (pooled samples). Thus, a value of 1.0 for a particular probe after normalization corresponds to the population average copy number and does not necessarily denote the usual copy dosage of 2 of a given locus. In order to estimate the copy number state of each locus in each individual, we developed a novel method that will be implemented in the R-package called MLPAstats [100]. In brief, since the MLPA dosage quotient ratios of the individuals at a certain locus are distributed according to a mixture of normal distributions, it is possible to assign to each individual a posterior probability of being located within one of the normal distributions that represent each copy number state. In order to assess if the copy number distribution was significantly different among populations, we performed a Fisher test after constructing contingency tables with copy number states and number of individuals assigned to each state. To determine the population in which a given CNV occurs, its distribution in each population was compared in a pairwise manner also using a Fisher's test (or χ^2^ test, depending on whether the number of individuals carrying a specific copy number exceeded a certain threshold).

### Expression data

Expression data for genes located within the identified CNVs was obtained from [38], available at ftp://ftp.sanger.ac.uk/pub/genevar/. We extracted the “globally” normalized information for unrelated individuals for whom we had estimated the CNV status. The existence of population-specific differences in expression levels of probes was determined by comparing the expression data using a Kruskal-Wallis test. In order to test for the relationship between copy number and expression levels we performed an ANOVA by fitting a linear model using the individuals' copy number estimated from MLPA and the expression data. Some of the genes located within CNV regions were not included in the analysis because their expression levels were below the average of negative control genes included in the array (bacterial genes *lysA*, *pheA*, *thrB* and *trpF*) across the different experiments (6.139), indicating that the hybridization to the array probes is below the level of detection.

### Gene ontology analysis

We used the Gene Ontology Tree Machine (GOTM - http://bioinfo.vanderbilt.edu/gotm/) to measure the enrichment in GO categories of the genes located within the CNVs showing population differences in comparison with the rest of genes of the human genome and the subset of genes located within known CNVs. In order to identify GO categories with significantly enriched gene numbers, the GOTM compares the distribution of genes in the user's set in each GO category to those in the reference gene set. GOTM reports only those enrichments that are statistically significant as determined by the hypergeometric test. Further details on the statistical background for the comparison between gene sets are given on the original publication [58].

### Selective constraints of genes located within CNVs

We obtained information on rates of synonymous (Ks) and non-synonymous (Ka) changes between humans and chimpanzees for genes located within the validated CNVs from Ensembl (http://www.ensembl.org/) and Khaitovich *et al.* [101]. To check for the existence of significant Ka/Ks differences of our gene set, we compared the average and median values with the values obtained from 10000 permutations of the same number of RefSeq genes. The *P*-value was calculated as the number of times that the Ka/Ks values of the permuted sets equaled or exceeded the observed Ka/Ks divided by the total number of permutations plus one [102]. Whole data set of Ka/Ks values used is provided in Tables S6 and S7.

### SNPs and CNVs association

We used HapMart to retrieve the genotypes of all HapMap SNPs (excluding monomorphic ones) in a window of 100 kb at each side of the estimated CNV breakpoints, or around the position of the MLPA probe that we used for the validation of single oligonucleotide probe. The SNPassoc R package [103] was then used to measure LD by calculating *r^2^* between the set of SNP genotypes and the copy number alleles. LD could only be calculated for biallelic copy number variants. In addition, in some cases, LD value could not be calculated because the CNV was identified exclusively in non-unrelated HapMap individuals or in individuals of Asian ancestry that were used in our analysis but have not been genotyped as part of the HapMap. Pairwise comparison of *r^2^* LD patterns among different populations was done using the Spearman's rank correlation coefficient.

### Evidences of natural selection on identified CNVs

For the tag-SNPs showing the highest linkage disequilibrium with the identified copy number variants in the population where the CNV was identified, we obtained the global and pairwise *F_ST_* values among populations for the Phase II HapMap SNPs from [84]. To detect population differentiation, we assumed a relatively strict threshold of global *F_ST_*≥0.2, and the comparison of pairwise *F_ST_* values (i.e. CEU vs YRI, CEU vs ASN, and YRI vs ASN) for a given tag-SNP pointed out which population was more clearly differentiated.

To test for signals of recent positive selection we compared, among populations, the integrated Haplotype Score (iHS) of the SNP that best tagged the variant in the population in which the CNV differences were found. Absolute values of iHS over 2 are generally accepted as signals of recent positive selection [86]. iHS data for HapMap II SNPs was retrieved from the Haplotter website (http://hg-wen.uchicago.edu/selection/index.html).

## Supporting Information

Table S1 Details of regions interrogated with the MLPA probes and summary of MLPA results obtained for each probe. Conservation was only explored in regions that were confirmed to be CNVs and did not contain genes. WGTP stands for Whole Genome Tiling Path Array.
(0.16 MB XLS)

Table S2 Details of MLPA probes used in the study. Genomic positions are referred to the hg18 UCSC genome assembly. MLPA probes are composed of two oligonucleotides that would only render an amplifiable product if properly ligated when bound to the specific DNA sequence. Right oligonucleotide sequences are entirely complementary to the target locus, while left oligonucleotide sequences are formed by the locus-specific sequence plus a stuffer sequence that would allow distinguishing each product in the mix. In the left sequence, stuffer sequence is lowercase and a dash separates it from the specific sequence, that is either upper or lowercase depending on the coincidence with a repeat identified by Repeat Masker. VIC, NED and FAM are the fluorophores that were used to label the specific universal primers combined in each probe mix.
(0.05 MB XLS)

Table S3 Comparison of CNVs identified in this study and previsouly existing information on the Database of Genomic Variants (DGV). The agreement column states whether our findings agree with previously published results (Agree), do not agree (Disagree), or could not be verified (Unverifiable) either because the CNV herein identified was not previously reported or there were inconsistencies in the description of the CNV in the original paper or in the Database of Genomic Variants.
(0.04 MB XLS)

Table S4 Gene Ontology Analysis (part I). Gene ontology analysis of genes located within the identified CNVs reveals enrichment in different functional categories when compared to the rest of human genes.
(0.02 MB XLS)

Table S5 Gene Ontology Analysis (part II). Gene ontology analysis of genes located within the identified CNVs reveals enrichment in different functional categories when compared to genes located within known CNVs.
(0.01 MB XLS)

Table S6 Comparison of Ka/Ks ratios of genes located within population-specific CNV changes. For the Ensembl dataset, mean and median values are calculated from dN/dS. For the calculations on the Khaitovich dataset, we used the Ka/Ki measure. P-values were calculated as the number of times that the observed mean value exceeded or equaled the value in the permutation analysis out of 10,000 permutations.
(0.02 MB XLS)

Table S7 Indexes of molecular evolution. All evolutionary measures were published or available elsewhere as described in the Methods section. Values were calculated by comparing human versus chimpanzee sequences. Ka is the number of non-synonymous nucleotide substitutions per non-synonymous site. Ks is the number of synonymous nucleotide substitutions per synonymous site. Ki is the number of substitutions per site in interspersed repeats in a 250-kbp window around the center of each gene (see Khaitovich et. al, 2005). dn/ds: number of non-synonymous substitutions per non-synonymous site divided by the number synonymous substitutions per silent site.
(0.02 MB XLS)

Table S8 Linkage disequilibrium for SNPs and CNVs. In the table it is reflected the calculated r-squared value (r2) and the SNP showing the maximum LD with the copy number state revealed by the MLPA probes in the first column. Values were obtained for each population independently and considering all populations together. The column SNPs indicates the number of HapMap SNPs retrieved for each analysed region flanking the CNV. For three of the probes it was not possible to measure LD because more than three copy number states were present (**) or because no SNP data was available for individuals showing the CNV (***).
(0.03 MB XLS)

Table S9 Haplotype structure correlation between populations. LD patterns correlation between populations was measured using the Spearman's rank correlation test. High Rho values indicate correlation and p-values below 0,05 denote significance for the correlation. For three of the probes it was not possible to measure LD because more than three copy number states were present (**) or because no SNP data was available for individuals showing the CNV (***).
(0.02 MB XLS)

Table S10 Evaluation of selective pressures on SNPs tagging CNVs. SNPs having the highest LD in the population in which the CNV variant was identified are shown in the table. We have recovered FST values calculated after pairwise population comparisons from Barreiro et al, (2008). The Inferred Pop column indicates to which population is attributable the differentiation, according to pairwise comparisons of the SNP FST. We used a strict criterion and assumed values above 0.20 to be indicative of population differentiation. iHS stands for integrated Haplotype Score (Voight et al, 2006) and absolute values higher than 2 indicate recent selection (marked with two asterisks). *: denotes coincidence of the most differentiated population with the population in which de CNV was identified. NA: indicates that we could not find Fst calculations or iHS value for the given SNP. None: indicates that we can not identify which is the differentiated population using the observed values. It is also used to indicate that no population has values above the iHS threshold considered for recent selection. **: indicates an absolute value of iHS above 2.
(0.07 MB XLS)

Figure S1 Evaluation of copy number in large CNVs. MLPA probes located within the same theoretical CNV do not show the same copy number pattern neither, in consequence, the same correlation with gene expression levels.
(0.01 MB PNG)

Figure S2 Correlation between copy number changes and expression levels. Each coloured circle corresponds to the measured copy number state plotted against expression levels for each HapMap individual. Gene name for which expression levels and copy numbers are depicted is shown in the title of each figure. Left panel (A) shows four examples of CNVs identified in which copy number is correlated with gene expression. Right panel (B) exemplifies four cases in which copy number differences exist among individuals from different populations, but it does not translate into gene expression differences.
(5.57 MB TIF)
