# Supplementary figures and images for: Identification of Copy Number Variants Defining Genomic Differences among Major Human Groups
Source: PLoS One. 2009 Sep 30;4(9):e7230. doi: 10.1371/journal.pone.0007230 (PMC2747275; doi:10.1371/journal.pone.0007230)

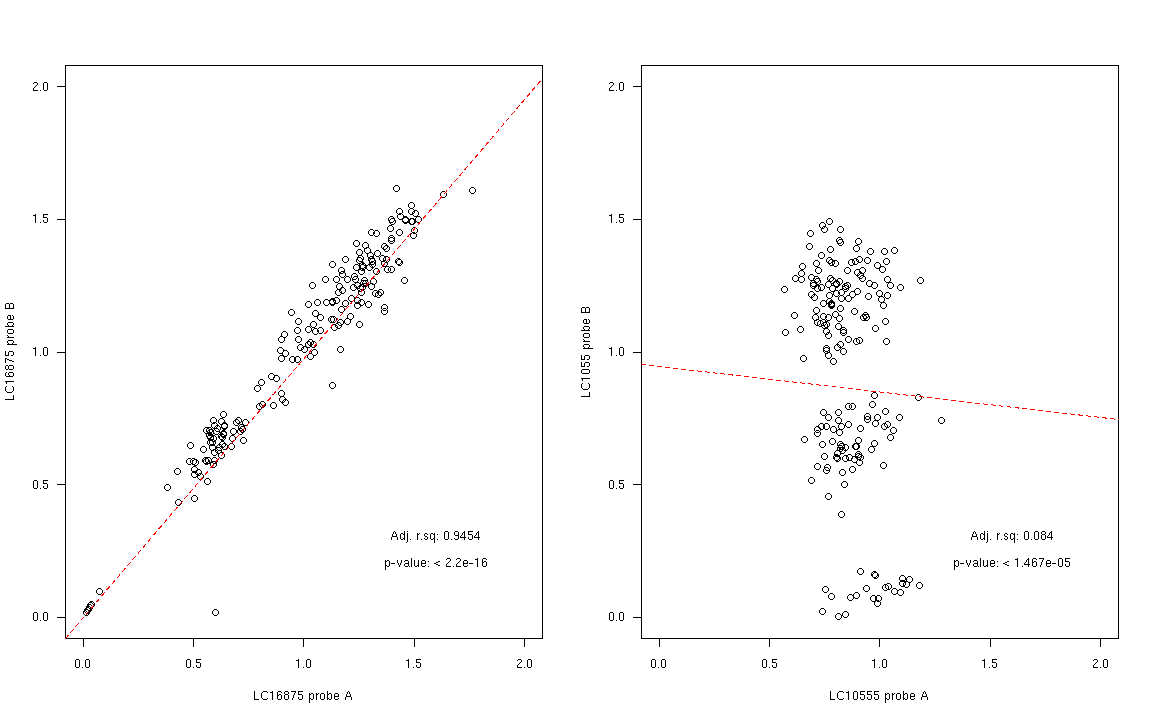

Supplement: Figure S1 — Evaluation of copy number in large CNVs. MLPA probes located within the same theoretical CNV do not show the same copy number pattern neither, in consequence, the same correlation with gene expression levels. (0.01 MB PNG) [file pone.0007230.s011.png]

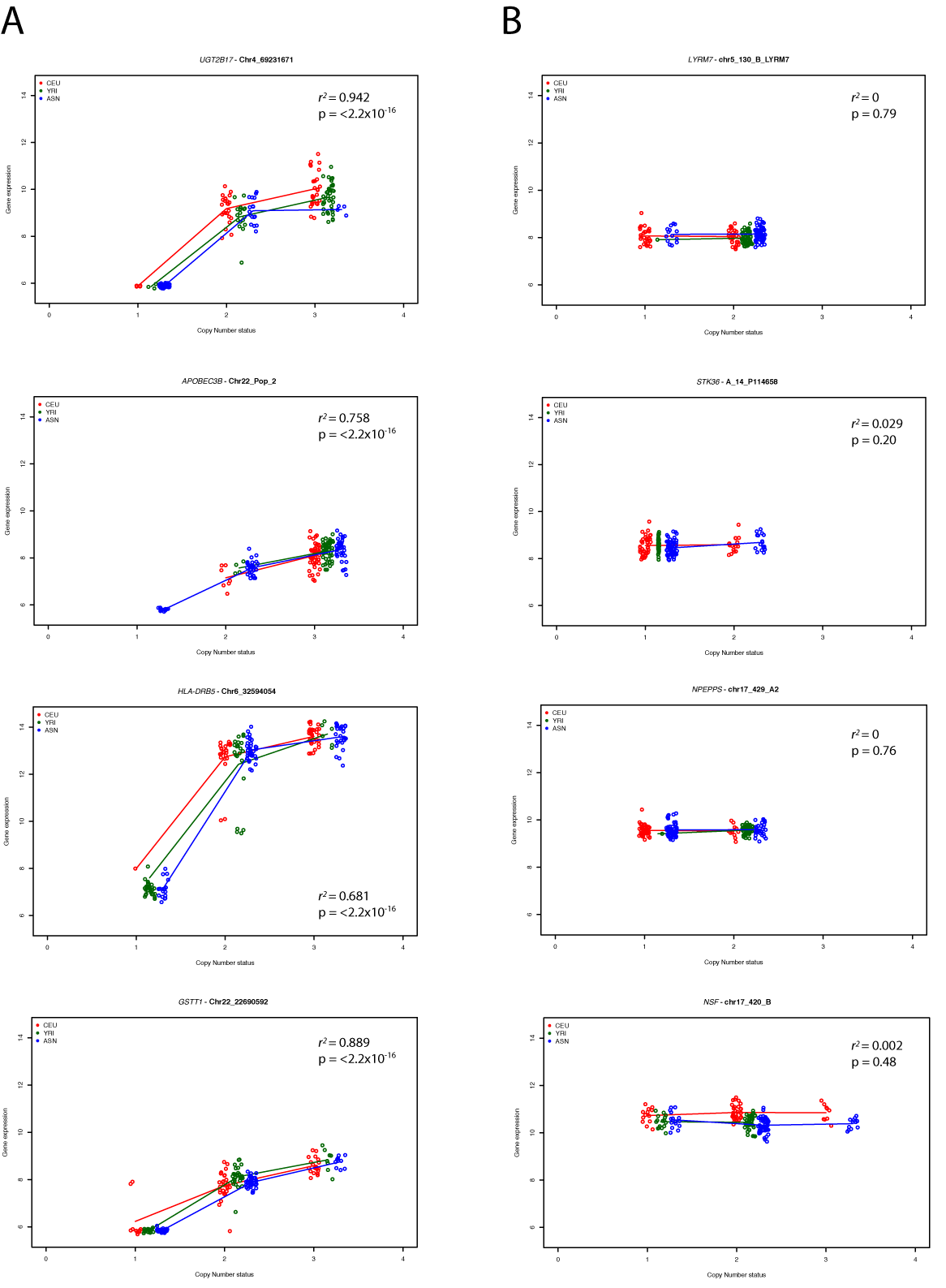

Supplement: Figure S2 — Correlation between copy number changes and expression levels. Each coloured circle corresponds to the measured copy number state plotted against expression levels for each HapMap individual. Gene name for which expression levels and copy numbers are depicted is shown in the title of each figure. Left panel (A) shows four examples of CNVs identified in which copy number is correlated with gene expression. Right panel (B) exemplifies four cases in which copy number differences exist among individuals from different populations, but it does not translate into gene expression differences. (5.57 MB TIF) [file pone.0007230.s012.tif]
